# Fine Mapping and Characterization of a Major Gene Responsible for Chlorophyll Biosynthesis in *Brassica napus* L.

**DOI:** 10.3390/biom12030402

**Published:** 2022-03-04

**Authors:** Chengke Pang, Wei Zhang, Menlu Peng, Xiaozhen Zhao, Rui Shi, Xu Wu, Feng Chen, Chengming Sun, Xiaodong Wang, Jiefu Zhang

**Affiliations:** 1 State Key Laboratory of Crop Genetics and Germplasm Enhancement, Nanjing Agricultural University, Nanjing 210095, China; pangcke@gmail.com (C.P.); pengmenlu1026@gmail.com (M.P.); haoba18893708851@gmail.com (X.Z.); s77990702@gmail.com (R.S.); 2 Key Laboratory of Cotton and Rapeseed, Ministry of Agriculture and Rural Afairs, Institute of Industrial Crops, Jiangsu Academy of Agricultural Sciences, Nanjing 210014, China; 20130032@jaas.ac.cn (W.Z.); xxdr5555@gmail.com (X.W.); 20030004@jaas.ac.cn (F.C.); suncm8331537@gmail.com (C.S.); 3 School of Food and Biological Engineering, Jiangsu University, Zhenjiang 212013, China

**Keywords:** *Brassica napus*, quantitative trait loci sequencing, candidate gene, chlorophyll deficiency, fine mapping, molecular marker

## Abstract

Rapeseed (*Brassica napus* L.) is mainly used for oil production and industrial purposes. A high photosynthetic efficiency is the premise of a high yield capable of meeting people’s various demands. Chlorophyll-deficient mutants are ideal materials for studying chlorophyll biosynthesis and photosynthesis. In a previous study, we obtained the mutant *yl1* for leaf yellowing throughout the growth period by ethyl methanesulfonate mutagenesis of *B. napus*. A genetic analysis showed that the *yl1* chlorophyll-deficient phenotype was controlled by one incompletely dominant gene, which was mapped on chromosome A03 by a quantitative trait loci sequencing analysis and designated as *BnA03.Chd* in this study. We constructed an F_2_ population containing 5256 individuals to clone *BnA03.Chd*. Finally, *BnA03.Chd* was fine-mapped to a 304.7 kb interval of the *B. napus* ‘ZS11’ genome containing 58 annotated genes. Functional annotation, transcriptome, and sequence variation analyses confirmed that *BnaA03g0054400ZS*, a homolog of *AT5G13630*, was the most likely candidate gene. *BnaA03g0054400ZS* encodes the H subunit of Mg-chelatase. A sequence analysis revealed a single-nucleotide polymorphism (SNP), causing an amino-acid substitution from glutamic acid to lysine (Glu1349Lys). In addition, the molecular marker BnaYL1 was developed based on the SNP of *BnA03.Chd*, which perfectly cosegregated with the chlorophyll-deficient phenotype in two different F_2_ populations. Our results provide insight into the molecular mechanism underlying chlorophyll synthesis in *B. napus*.

## 1. Introduction

Rapeseed (*Brassica napus* L., AACC, 2n = 38) is an important source of vegetable oil worldwide and is also used to produce biodiesel, ethanol, lubricant oil, and other industrial goods [1]. Compared to traditional diesel, biodiesel produces less greenhouse gases, and the energy per unit volume of biodiesel is higher [2]. Through the improvement of traditional rapeseed varieties, the cake of double-low rapeseed (low erucic acid and low glucosinolate content) can also be used to produce livestock feed [3]. The use of rapeseed oil for nonfood applications has increased rapidly since 2000 [1]. Therefore, under the background of limited arable high-quality land resources, researchers need to continuously increase rapeseed yield per unit land area to meet the demand for industrial and edible oils. Photosynthesis is the most important chemical reaction on earth, and a high crop yield requires a high photosynthetic efficiency. It is of great significance to study plant photosynthesis to improve photosynthetic efficiency.

In *Arabidopsis*, there are 15 enzymes encoded by 27 genes involved in the biosynthesis of chlorophyll [4]. Any gene mutation causing a blockage in chlorophyll synthesis and, hence, pigment ratio changes can result in phenotypes with varying degrees of green color deficiency [5,6]. Various leaf color mutants have been obtained through mutation, gene editing, and other methods in *Arabidopsis* [7], rice [8], maize [9], soybean [10], and wheat [11]. Some leaf color mutants have also been isolated in *B. napus* [12,13]. The inhibiting expression of the gene encoding glutamate-1-semialdehyde aminotransferase in the chlorophyll synthesis pathway can affect the chlorophyll content of *B. napus* seeds [14]. If the expression of the chlorophyll *a* oxygenase gene was blocked during chlorophyll synthesis in *Brassica rapa* ssp. *pekinensis*, there would be a chlorophyll-deficient phenotype [15]. Zhao et al. isolated a yellow-virescent leaf (*yvl*) mutant in *B. napus*, and the gene encoded H subunit of magnesium chelatase (MgCH) was considered the most likely candidate gene responsible for the *yvl* phenotype [16]. A single-nucleotide polymorphism (SNP) generated a premature stop codon, which impaired the protein structure and reduced the activity of the H subunit of MgCH (ChlH) [16].

Previous research had established that MgCH catalyzes the conversion of protoporphyrin IX (Proto IX) to Mg-Proto IX, which is the first step unique to chlorophyll synthesis [17]. MgCH is a large, multisubunit complex. The catalytic reaction needs cooperative action between the three subunits of MgCH: the catalytic subunit ChlH, where the insertion of Mg^2+^ takes place, and two AAA^+^ subunits (ChlI and ChlD) [17,18]. The function of the three subunits has been studied in several crops, such as rice [19], soybean [20], and *Arabidopsis* [21,22]. The crystal structure of ChlH of photosynthetic cyanobacterium *Synechocystis* PCC 6803 includes six domains (I–VI). The active site of ChlH is buried inside the protein interior and the surrounding amino acid residues are conserved [17]. Mutations in genes encoding any subunit of MgCH may cause chlorophyll synthesis to be blocked [19,21]. Most chloroplast proteins are encoded by nuclear genes, and a few are encoded and regulated by cytoplasmic genes. The signal transduction between the nucleus and chloroplast affects the synthesis of chlorophyll. Genomes uncoupled (GUN) loci have been identified as components of the plastid-to-nucleus signal transduction in *Arabidopsis*. Mochizuki et al. (2001) cloned the *GUN5* gene in *Arabidopsis* and showed that it encodes ChlH [21]. A mutation at a conserved site of ChlH (Ala990Val) caused a genomes uncoupled phenotype [17,21]. Therefore, ChlH not only participates in the catalysis as a subunit of MgCH, but also plays an important role in the plastid-to-nucleus signal transduction. At the same time, ChlH is an abscisic acid (ABA) receptor, which specifically binds ABA and regulates seed germination, postgermination growth, and stomatal movement [23]. The GUN4 protein activates MgCH by binding with the ChlH [24].

In addition to those enzymes directly involved in the chlorophyll synthesis pathway, other pathways also affect chlorophyll synthesis and, hence, the chlorophyll content. The chlorophyll intermediate Mg-Proto IX accumulates and negatively regulates the expression of photosynthetic genes under stress conditions [25,26]. At the same time, from 5-aminolevulinic acid (ALA) to Proto IX, there is a common precursor for chlorophyll and heme biosynthesis. Proto IX is located at the branch point of heme and chlorophyll biosynthesis. Therefore, the impairment of heme and other pathways also affects the biosynthesis of chlorophyll by multiple regulatory mechanisms. An overaccumulation of heme is known to inhibit the activity of glutamyl-tRNA reductase (GluTR) in a feedback manner [27]. For example, mutations in the gene encoding heme oxygenase 1, involved in heme breakdown, showed a chlorophyll-deficient phenotype in the *B. napus* mutant *ygl* [28]. The transcription of plastid genes was performed by two types of RNA polymerase in higher plants, plastid-encoded polymerase (PEP) inherited from their cyanobacterial ancestor, and nuclear-encoded polymerase (NEP). Many photosynthesis-related genes are transcribed by PEP (class I), whereas some genes are only transcribed by NEP (class III). On the other hand, many other housekeeping genes are transcribed by both PEP and NEP (class II) [29]. PEP activity affects chloroplast biogenesis and photosynthesis [30]. Compared to other crops, there are still few published studies on leaf color mutants in *B. napus*, with only a few of the corresponding genes having been identified. *B. napus* is a tetraploid species, which originated from a hybridization between *B. rapa* (AA, 2n = 20) and *B. oleracea* (CC, 2n = 18) around 7500 years ago. The publication of the “Darmor-*bzh*” genome has greatly promoted the study of gene cloning and function in *B. napus* [31]. In previous studies, we obtained the mutant *yl1* with a pale-green phenotype. Compared to NY18, the average plant height, branch height, and 1000-seed weight of *yl1* decreased by ~20%, 60%, and 34.5%, respectively. At the seedling stage, the contents of chlorophyll *a*, chlorophyll *b,* and the carotenoids of *yl1* also significantly decreased [32]. The present study was designed to fine map the gene responsible for the chlorophyll deficiency phenotype in mutant *yl1*, using the quantitative trait loci sequencing (QTL-Seq) analysis and map-based cloning strategies. A functional marker, based on the target gene, was developed which cosegregated with the chlorophyll-deficient phenotype. This work promotes plant breeding based on molecular marker-assisted selection (MAS) breeding and the exploration of molecular mechanisms that regulate chlorophyll biosynthesis in *B. napus*.

## 2. Materials and Methods

### 2.1. Plant Materials

The chlorophyll-deficient mutant *yl1* was isolated from ethyl methanesulfonate (EMS)-mutagenized lines of a conventional *B. napus* variety Ningyou 18 (NY18) bred by Jiangsu Academy of Agricultural Sciences, Nanjing, Jiangsu Province, China [32]. The mutagenic process of materials followed the description of Wang et al. [33]. Mature seeds of NY18 were soaked in 1.0% EMS solution (*w*/*v*, Sigma-Aldrich, St. Louis, MO, USA) for 12 h at 25 °C in pH 7.0 phosphate buffer. The mutagenized seeds were sown in the field after being rinsed with water for 4 h. The characters of mutagenized individuals were monitored throughout the entire growth period, and individual plants with chlorophyll deficiency were bagged at the flowering stage. The chlorophyll-deficient mutant *yl1* was propagated by multiple generations of self-pollination and was shown to be stably inherited.

The (NY18 × *yl1*) F_2_ population, derived from a cross between NY18 and *yl1* containing 264 individual lines, was used for QTL-Seq [34]. The (Holly × *yl1*) F_2_ population, which contained 5256 individuals, was used as a segregating population for fine mapping of the candidate gene associated with the chlorophyll-deficient phenotype. The (ZS11 × *yl1*) F_2_ population derived from ZS11 crossed with *yl1*, which contained 368 individuals, and the (NY18 × *yl1*) F_2_ lines were used to verify the effectiveness of functional molecular markers. All individual plants were grown in the experimental plot of Jiangsu Academy of Agricultural Sciences.

### 2.2. Quantitative Trait Loci Sequencing Analysis

The leaf color of individual plants of the (NY18 × *yl1*) F_2_ population was observed at the seedling stage, and the SPAD value was measured using a SPAD-502 m (Konica-Minolta, Tokyo, Japan). The previous statistical results showed that the SPAD value of wild-type plants was >20, the SPAD value of the homozygous mutant plants was <10, and the heterozygous value was between 10 and 20 [32]. The phenotypic characteristic was determined according to the SPAD value of the single plant. The young leaves of 25 wild-type lines (G) and 25 homozygous mutant lines (Y) were collected and stored at −80 °C. Plant Genomic DNA Kit (TIANGEN, Beijing, China) was used to extract genomic DNA. The genomic DNA G-pool and Y-pool were constructed by mixing equal proportions of the respective individual DNAs. The purity and integrity of DNA were analyzed by spectrophotometry with a Nanodrop 2000 instrument (Thermo Fisher Scientific, Waltham, MA, USA) and agarose gel electrophoresis, respectively.

The resequenced data of the two parents and the two F_2_ bulks were generated using the Illumina HiSeq™ PE150 (Illumina, Inc., San Diego, CA, USA) platform by Novogene Bioinformatics Technology Co., Ltd. (Beijing, China). To make sure reads were reliable and without artificial bias in the following analyses, raw reads of fast format were firstly processed through a series of quality control (QC) procedures in-house C scripts. QC standards were as follows: (1) removing reads with ≥10% unidentified nucleotides, >50% bases having phred quality <5; (2) removing reads with >10 nt aligned to the adapter, allowing ≤10% mismatches; (3) removing putative PCR duplicates generated by PCR amplification in the library construction process. Clean reads were aligned against the reference genomes of “ZS11” [35] and “Darmor-*bzh*” [31] using Burrows-Wheeler Aligner (BWA) software [36] and repetitive read pairs were removed using the Sequence Alignment Map tools [37]. The SNPs in all the samples were detected using the Unified Genotyper module of GATK software and filtered using the Variant Filtration parameter [38]. To identify candidate regions associated with the chlorophyll-deficient trait, the SNP-index and Δ(SNP-index) were calculated for all genomic positions in the G-pool and Y-pool, using the genotype of NY18 as the reference. SNP-index values were calculated in sliding windows of 1 Mb with a 10 Kb step and the average SNP-index value for each window was recorded along the chromosome. The Δ(SNP-index) = SNP-index (Y-pool) − SNP-index (G-pool) [33].

### 2.3. Fine Mapping of Candidate Genes

Penta-primer amplification refractory mutation system (PARMS) technology is a newly developed SNP genotyping method based on fluorescence detection [39]. After identifying the candidate interval for the target gene by QTL-Seq, PARMS SNP markers were used to screen recombinant plants for fine mapping among the (Holly × *yl1*) F_2_ population, with total DNAs of (Holly × *yl1*) F_2_ individuals being extracted from fresh rapeseed leaves using cetyltrimethylammonium bromide method [40].

According to the parental resequencing information, homozygous SNPs were selected to design PARMS SNP markers. The 200 bp sequences upstream and downstream of the selected SNP (401 bp in total) were submitted to the SNP automatic typing tool (http://www.snpway.com/ (accessed on 29 April 2019)) to design the primers for PARMS SNP markers (Supplementary Table S1). The master mix for PARMS markers was obtained from Gentides Biotech Co., Ltd. (Wuhan, China). First, the SNPs at each end of the candidate interval were used to develop two PARMS molecular markers. These two markers were used to screen individuals of the (Holly × *yl1*) F_2_ population for the chlorophyll-deficient phenotype. Plants combining yellow leaf color and low chlorophyll content (expressed as SPAD value), with exchange recombination between the two markers, were identified by screening. The recombinant plants were then analyzed with the newly developed polymorphic PARMS SNP markers.

### 2.4. RNA Library Construction and RNA-Seq Analysis

Equivalent weights of leaves from wild-type NY18 and mutant *yl1*, with three biological replicates each, were collected at the seedling stage. RNA library construction and sequencing followed the description of Wang et al. [33]. RNA-Seq was performed on an Illumina HiSeq 2000 platform by Novogene Bioinformatics Technology Co., Ltd. (Beijing, China). The reads containing adapters, poly-Ns, and low-quality reads among the raw reads from RNA-Seq were processed using Trimmomatic version 0.36 [41]. The sequencing quality was evaluated by GC content, base mass value (Q20, Q30), and other indicators. Then, clean reads were mapped to the reference genome “Darmor-*bzh*” [26] using TopHat version 2.0.6 [42]. The distribution of reads on each sample transcript was calculated to evaluate the randomness of RNA-Seq using the RNA-Seq Quality Control package (RSeQC) [43]. Differential expression analyses were carried out to locate differentially expressed genes (DEGs), with a false discovery rate < 0.05 and |log_2_fold change| ≥ 1, using the DESeq R package (1.10.1). To discern the functions of DEGs, DEGs were submitted to the Kyoto Encyclopedia of Genes and Genomes (KEGG) website (https://www.genome.jp/kegg/ (accessed on 1 June 2018)), and the KEGG of DEGs was performed using the KOBAS online analysis database [44].

### 2.5. Gene Cloning and Structure Prediction

To further study the causes of chlorophyll deficiency in mutant *yl1*, we performed sequence analysis on candidate genes. According to the *B. napus* “ZS11” reference genome, primers were designed to clone the cDNA sequences of the putative candidate genes by Primer Premier version 5.0 (Table 1). PCR amplification was performed according to the procedures shown in Supplementary Table S2. The PCR products were sequenced by Novogene Bioinformatics Technology Co., Ltd. (Beijing, China). The cDNA and protein sequence alignments were performed using DNAMAN software (https://www.lynnon.com/dnaman.html (accessed on 21 June 2019)). The mutated sequence was submitted to the Phyre2 web portal for protein prediction and analysis [45].

### 2.6. Gene Expression Analysis by Quantitative Real-Time PCR (qRT-PCR)

To study the expression of candidate genes in various tissues, we took the second leaf at three-leaf stage, the third leaf at five-leaf stage, the upper stem at bolting stage, and the silique shell at pod-formation stage for qRT-PCR. SYBR Green I was used as the fluorescent dye, and the reference gene was the *Actin-7* (GenBank: AF111812.1) gene [46]. Primers (Table 1) were designed using the Primer Premier 5.0 program [47]. The qRT-PCR was performed on the ABI-7500 fluorescence quantitative PCR instrument (Applied Biosystems, Foster City, CA, USA). The 2^−ΔΔCt^ method was used to calculate the relative expression levels of the candidate gene in different tissues [48].

### 2.7. Developing Molecular Marker

To utilize the chlorophyll-deficient mutant *yl1*, a PARMS molecular marker was designed based on the SNP of the candidate gene and named BnaYL1, using the SNP decoder tool (http://www.snpway.com/snp-decoder01/ (accessed on 12 August 2019)). The universal primers that were consistent with the latter half sequences of the two forward primers had different fluorescent marks at the tail, with FAM/HEX universal fluorescent primers preset in the 2× PARMS Master Mix (Gentides, Wuhan, China). The synthesis of primer and marker detection was carried out by Gentides Biotech Co., Ltd. (Wuhan, China).

### 2.8. Statistical Analysis

The statistical analyses of the results of qRT-PCR in this study were performed by one-way analysis of variance (ANOVA) and Fisher’s least significant difference (LSD) test. The number of biological repeats was annotated in each experiment.

## 3. Results

### 3.1. Phenotypic Characterization and Genetic Analysis

**Figure 1:**
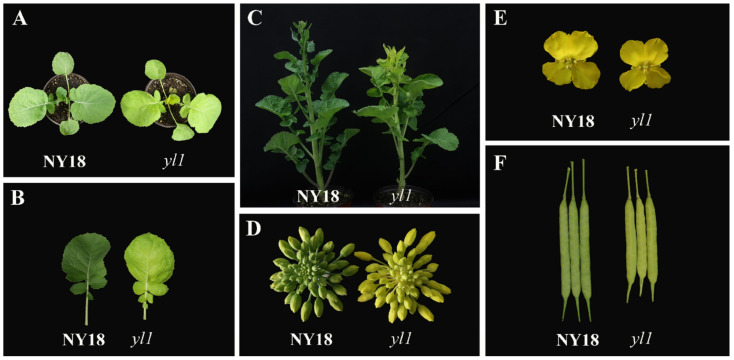
Phenotypic expression of mutant *yl1* over the entire growth period. (**A**) The phenotype of NY18 and *yl1* in seedling stage; (**B**) leaf color of NY18 and *yl1*; (**C**) the phenotype in budding stage; (**D**) the color of flower buds in NY18 and *yl1*; (**E**) the size of petals; (**F**) the color and size of siliques.

The *yl1* gene mutation resulted in the chlorophyll deficiency of the mutant plants throughout the entire growth period. Compared with wild-type NY18, the leaves of *yl1* were significantly yellowed at the seedling stage (Figure 1A,B). As the plants grew, the cotyledons still showed the chlorophyll-deficient phenotype, but the color of the more mature leaves turned slightly greener (Figure 1C). The above phenotypic characteristics were described in another article in our laboratory [32]. At the same time, the flower buds and siliques, the main photosynthetic organ after the flowering of *yl1*, also showed the obvious chlorophyll-deficient trait (Figure 1D,F). The size of the flowers and siliques in *yl1* was significantly smaller than those in NY18 (Figure 1E,F). The plant height and 1000-seed weight were significantly higher in NY18 than in *yl1* [32].

### 3.2. Initial Localization of Candidate Genes

Genomic DNA from NY18, *yl1*, and the two F_2_ bulks (G- and Y-pools) was sequenced, and generated clean reads amounting to 100.7 Gb. The sequencing quality was high (Q20 ≥ 95.28%, Q30 ≥ 88.52%). The reads were aligned to the *B. napus* ‘ZS11’ reference genome, and the comparison rate for all samples was between 97.25% and 98.52% (Supplementary Table S3). The depths of sequencing coverage for the G-pool, Y-pool, NY18, and *yl1* were 39.82-fold, 33.75-fold, 16.79-fold, and 17.88-fold, respectively. The coverage of 1× (at least one base) was more than 92.89% (Supplementary Table S3). All samples had sufficient data, the GC distribution was normal, and the library sequencing was successful, allowing it to be used for subsequent mutation detection.

**Figure 2:**
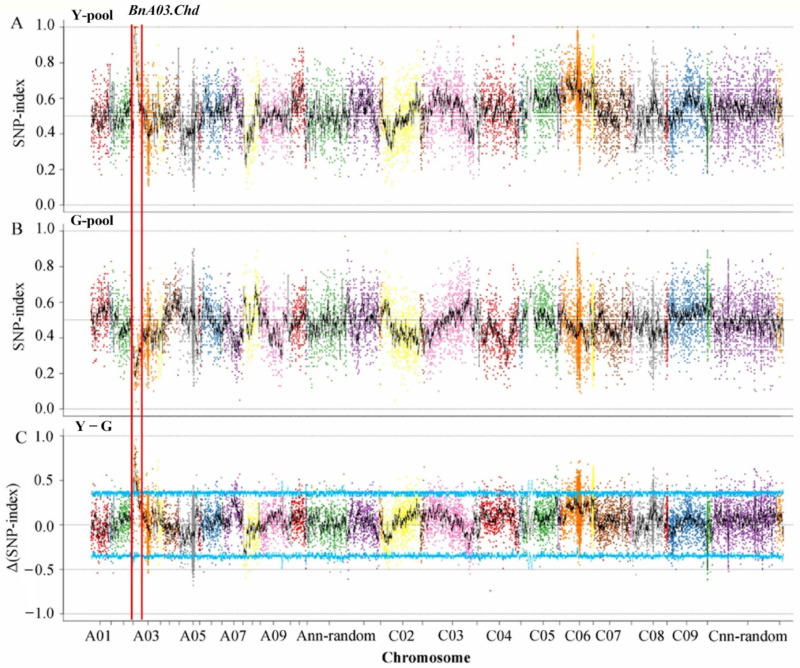
SNP-index and Δ(SNP-index) graphs from the QTL-Seq analysis. SNP-index of (**A**) Y-pool (homozygous mutant leaves) and (**B**) G-pool (wild-type leaves); (**C**) Δ(SNP-index) of Y-pool and G-pool. The *BnA03.Chd* on chromosome A03 was regarded as the candidate interval, with an α = 0.05 significance level.

Based on the results of genotyping, 17,734 polymorphic SNPs with homozygous differences between the two parents were identified by screening after filtration. The SNP-indexes of the two pools on chromosome C06 distributed in the range of 0–1, and there was no case where the SNP-index values of the two pools were close to 1 and 0, respectively, due to the linkage with the target gene. The main gene heritability of F_2_ generation was 96.42% [32]. The genomic region from 1.50 to 5.19 Mb on chromosome A03, designated as *BnA03.Chd*, had an average Δ(SNP-index) of 0.64 at a α = 0.05 significance level, suggesting that this region included a major gene for the chlorophyll-deficient phenotype (Figure 2).

### 3.3. Fine Mapping of the BnA03.Chd Gene

**Figure 3:**
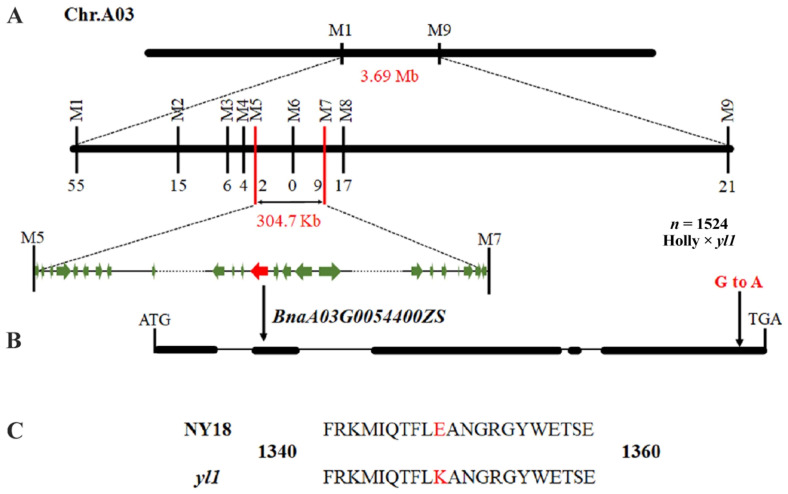
Fine mapping of the *BnA03.Chd* gene in (Holly × *yl1*) F_2_ population. (**A**) The *BnA03.Chd* locus was fine-mapped in a 304.7 kb interval containing 58 genes using 1524 chlorophyll-deficient individuals. (**B**) The *BnaA03G0054400ZS* was the final candidate gene and had an SNP (G to A) of the gene. (**C**) The SNP caused the change of glutamic acid at 1349th position to lysine.

The leaf color phenotype was judged by the SPAD value, and 1524 chlorophyll-deficient individuals with a SPAD value of less than 10 from the (Holly × *yl1*) F_2_ segregation population were selected at the seedling stage for fine mapping. First, two flanking PARMS SNP markers (Supplementary Table S1), M1 (physical position of the *B. napus* ZS11 chromosome A03: 1,499,657 bp) and M9 (5,194,567 bp), were designed to identify recombinants in the chlorophyll-deficient individuals (Figure 3). The results showed that there were 76 recombinant individuals within the chlorophyll-deficient population, of which 55 recombinant plants were detected in M1 and 21 in M9 (Supplementary Table S4). Then, based on the resequencing information of NY18 and *yl1*, markers M2 and M8 were designed and used for genotyping the 76 recombinant individuals. The results showed that markers M2 and M8 detected 15 and 17 recombinant plants, respectively. Next, marker M7 on the left-hand side of M8 was designed and obtained nine recombinant plants among the 76 recombinants. Subsequently, M3, M4, M5, and M6 markers between M2 and M7 were designed, which identified six, four, two, and zero recombinant plants, respectively. The marker M6 completely cosegregated with the chlorophyll-deficient trait. Finally, a 304.7 kbp interval between the M5 and M7 markers was determined as the final candidate interval (Figure 3A).

### 3.4. Genome-Wide Transcriptomic Analyses of NY18 and yl1

**Figure 4:**
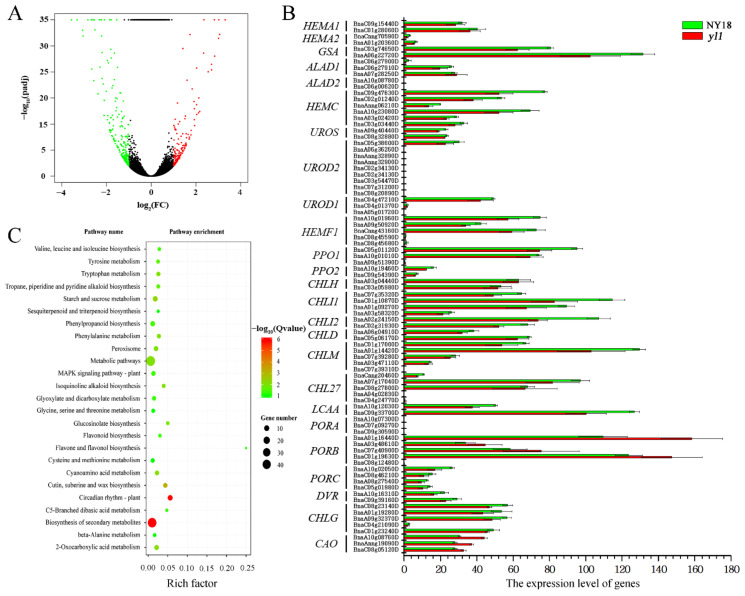
(**A**) Differentially expressed genes (DEGs) between *yl1* and NY18. (**B**) The expression level of genes involved in chlorophyll biosynthesis pathway. (**C**) KEGG pathway categories of differentially expressed genes between NY18 and *yl1* at leaves from the seedling stage. The size of the point represents the number of genes, and the color of the point represents the degree of enrichment.

After filtering out the low-quality reads, a total of 19.41–23.87 million clean reads for each sample was obtained through high-throughput RNA-Seq (Supplementary Table S5) and aligned to the “Darmor-*bzh*” reference genome [31]. The results showed that 146 (39.04%) of the genes were significantly upregulated and 228 (60.96%) of the genes were significantly downregulated in the yellow-leafed mutant *yl1*, compared with its isogenic parent, NY18 (Figure 4A).

Compared to NY18, *yl1* had significantly lower levels of chlorophyll *a* and chlorophyll *b*. There were 15 enzymes encoded by 27 genes involved in the biosynthesis of chlorophyll in *Arabidopsis*, and a total of 85 *B. napus* genes was homologous to the 25 of 27 genes (except *HEMA3*, *GSA2,* and *HEMF2*) (Figure 4B, Supplementary Table S6). Among the 85 genes, there were significant differences in the expression levels of different genes, and 12 of them were not expressed at all in either NY18 or *yl1* based on RNA-seq data. However, there was no significant difference in the expression of all 85 genes between NY18 and *yl1*. In *Arabidopsis*, 5-aminolevulinate dehydratase was encoded by *ALAD1* and *ALAD2*, but *ALAD1* was the major contributor at this step [49]. In normal circumstances, *PORA* mRNA disappeared within the first 4 h of greening [50]. The chloroplasts of *yl1* were abnormal and underdeveloped, and the thylakoids were loosely distributed [32]. We collected 44 *B. napus* genes, which were homologous to the light-harvesting chlorophyll *a/b*-binding proteins of the photosystem genes in *Arabidopsis* (Supplementary Table S7). Surprisingly, only one homolog (*BnaA02g29010D*) was significantly differentially expressed between NY18 and *yl1*.

The up- and down-regulated genes were enriched with respect to 14 and 16 pathways, respectively (Supplementary Table S8). KEGG enrichment results showed that there were more DEGs enriched in the biosynthesis of secondary metabolites, metabolic pathways, and circadian rhythm (Figure 4C). The *Golden 2-like* (GLK) transcription factor is one of a pair of partially redundant nuclear transcription factors that regulate chloroplast development in a cell-autonomous manner in *Arabidopsis* [51]. Compared with NY18, the expression of homologous genes of GLK decreased significantly in *yl1*. The LHC super-gene family encodes the light-harvesting chlorophyll *a/b*-binding (LHC) proteins, and *Lhcb2.4* was markedly down-regulated in *yl1* (Supplementary Table S7). On the contrary, chlorophyllase 2, the first enzyme in chlorophyll degradation, increased significantly in *yl1* (Supplementary Table S7).

### 3.5. Identification of a Candidate Gene

According to the gene annotations of the “ZS11” reference genome [35], there were 56 genes in the *BnA03.Chd* interval after removing two homologous genes (Supplementary Table S9). RNA-Seq results showed that none of the genes in the *BnA03.Chd* region were significantly differently expressed between NY18 and *yl1*. The resequencing alignment results showed that 15 genes had mutations in exons, among which 2 genes had frameshift deletion mutations, 3 genes had nonsynonymous mutations, and 10 genes had synonymous mutations. According to the principle of EMS mutagenesis, the mutation types were mostly of a single-base transition or transversion mutation, but *BnaA03G0052800ZS* and *BnaA03G0052900ZS* genes had frameshift deletion mutations. Moreover, the annotated information of the genes with a deletion mutation showed there was no correlation with chlorophyll synthesis, so *BnaA03G0052800ZS* and *BnaA03G0052900ZS* were excluded. The resequencing results showed that there was no expression of *BnaA03G0055600ZS* and *BnaA03G0056500ZS* with nonsynonymous mutants in NY18 or *yl1*, suggesting that the two genes could not be candidate genes for the chlorophyll-deficient trait. Therefore, the only remaining gene *BnaA03G0054400ZS* was preliminarily identified as the candidate gene for the chlorophyll-deficient trait.

Previous studies in *B. napus* and *Arabidopsis* have shown that the gene encodes ChlH and is evolutionarily conserved [16,21]. *BnaA03G0054400ZS*, a homolog of *AT5G13630*, encodes the H subunit of MgCH, which is heavily involved in chlorophyll biosynthesis and chloroplast development. MgCH initiates the chlorophyll biosynthetic pathway by inserting Mg^2+^ into the protoporphyrin macrocycle. ChlH is the insertion site of magnesium ions by Mg-chelatase.

### 3.6. Sequence Analysis of the Candidate Gene for BnA03.Chd

**Figure 5:**
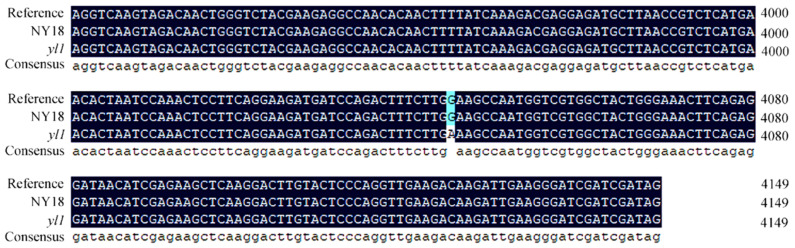
Sequence alignment of the coding sequence of *BnaA03G0054400ZS* gene between NY18 and *yl1*. There was a base substitution (G to A) at 4045 bp of the CDS in the fifth exon in *yl1*.

Based on the reference sequence of *BnaA03G0054400ZS*, we designed primers (Table 1) to amplify the cDNA of the isogenic wild-type NY18 and mutant *yl1*. The sequencing results of amplification products showed that the full-length of the coding sequence (CDS) of *BnaA03G0054400ZS* was 4149 bp. DNA sequence alignment showed that there was no difference in the promoter sequences between the two lines, but that there was a base substitution (G to A) at 4045 bp of the CDS in the fifth exon in *yl1* (Figure 5). The amino acid at the 1349th position was transformed from glutamic acid to lysine (i.e., E to K) (Supplementary Figure S1). The results of structure prediction showed that the secondary structure of the site did not change after mutation (Supplementary Figure S2A). The mutation site was located in the domain VI of ChlH (Supplementary Figure S1) and not in the proto-binding pocket of ChlH (Supplementary Figure S2B).

### 3.7. Expression Analysis of the Candidate Genes

**Figure 6:**
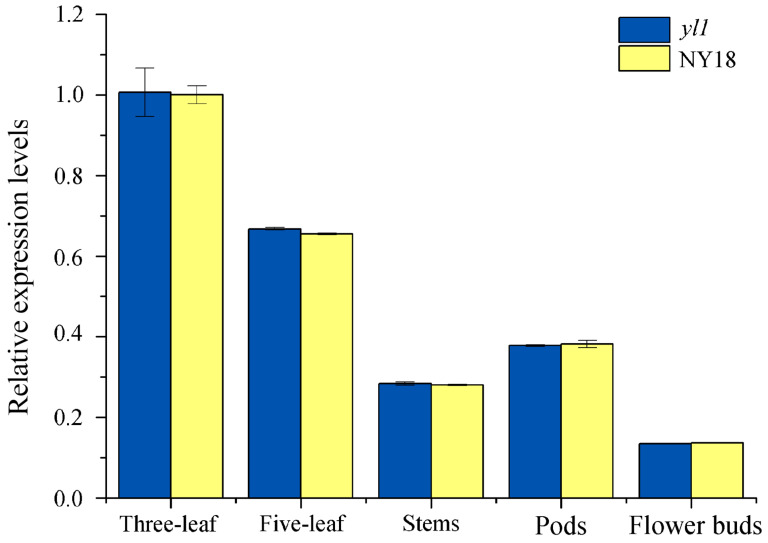
The expression level of *BnaA03G0054400ZS* in leaves, stems, pods, and flower buds of NY18 and *yl1*, as determined by quantitative real-time PCR (qRT-PCR). The values are presented as the mean ± SD (*n* = 3). The statistical analyses in this study were performed using one-way analysis of variance (ANOVA) and Fisher’s least significant difference (LSD) test.

The results of RNA-seq showed that the transcription level of *BnaA03G0054400ZS* in leaves of both NY18 and *yl1* had no significant difference (Supplementary Table S9). To further investigate the specific gene expression pattern, the expression level of *BnaA03G0054400ZS* in the leaf, stem, flower bud, and silique shell of NY18 and *yl1* was analysis by qRT-PCR (Supplementary Table S10). The results showed that the expression level of *BnaA03G0054400ZS* in all tested tissues of *yl1* was not significantly different from that of NY18 (Figure 6). In *B. napus*, leaves are the main photosynthetic organs before full flowering, and the expression of the *BnaA03G0054400ZS* gene was the highest in leaves. In nonmajor photosynthetic tissues such as the stem and bud, the expression level of *BnaA03G0054400ZS* was relatively low (Figure 6). This was consistent with the changing trend of expression levels of the candidate gene in various organs of ZS11 in the transcriptome database of *B. napus* (http://yanglab.hzau.edu.cn/BnTIR/expression_show (accessed on 10 January 2022)).

### 3.8. Development of the PARMS Marker for BnA03.Chd

**Figure 7:**
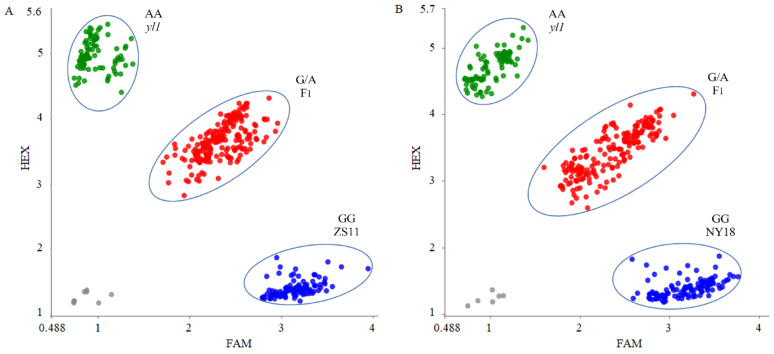
Genotyping results using marker BnaYL1 in (**A**) (ZS11 × *yl1*) F_2_ and (**B**) (NY18 × *yl1*) F_2_ segregation populations. The blue dot represents homozygous alleles derived from parent lines with normal chlorophyll content, the green dot represents homozygous alleles derived from parent line “*yl1*” with low chlorophyll content, and the red dot represents heterozygous loci. The grey dot represents negative control.

Based on the SNP of the candidate gene *BnaA03G0054400ZS*, we designed a PARMS marker, BnaYL1 (Table 1). Subsequently, the effectiveness of this marker was verified in two different populations. The (ZS11 × *yl1*) F_2_ population contained 368 individuals, in which fluorescence signals could be detected by BnaYL1 in 366 of the 368 samples. FAM fluorophore could be detected in 94 samples, and the plants corresponding to these samples showed the wild-type phenotype. FAM/HEX fluorophore signals were detected in 195 samples, and the corresponding plants exhibited the heterozygote phenotype. HEX fluorophore signals were detected in 77 samples, which corresponded to the homozygous mutant phenotype (Figure 7A). The marker BnaYL1 cosegregated perfectly with the chlorophyll-deficient phenotype in the (ZS11 × *yl1*) F_2_ population. In the individual plants in the (NY18 × *yl1*) F_2_ population, 107 wild-type homozygotes, 183 heterozygotes, and 83 mutant homozygotes were detected by BnaYL1, a finding which was completely consistent with the phenotypes (Figure 7B). The results showed that BnaYL1 had a high genotyping efficiency and could be used for MAS in the future. In addition, BnaYL1 cosegregated with the chlorophyll-deficient phenotype in both the (ZS11 × *yl1*) F_2_ and (NY18 × *yl1*) F_2_ populations, providing further proof that the *BnaA03G0054400ZS* gene is a potential candidate gene for *yl1*.

## 4. Discussion

*B. napus* is an important source of vegetable oil and biofuel in the world. However, in recent years, the improvement of rapeseed yield has encountered bottlenecks, and new technological progress is urgently needed to improve rapeseed yield. Efficient photosynthesis is an important factor in achieving a high yield in crops.

So far, all genes encoding chlorophyll synthesis in *Arabidopsis* have been cloned [52]. Many leaf color genes have been mapped in crops, such as rice [5,53,54], maize [9,55], and others [10,56]. However, there have still been relatively few studies on leaf color control sites in *B. napus*, with only a few leaf color genes being cloned [14,57,58]. Compared with other crops, the main photosynthetic organs switch from the leaves to pods in *B. napus*, from about two weeks after full flowering [59]. If the chlorophyll synthesis is blocked in leaves and pods, the yield of rapeseed is significantly reduced. In a previous study, we obtained a new chlorophyll-deficient mutant of *B. napus* by EMS mutagenesis. The leaves and siliques of this mutant showed a chlorophyll-deficient phenotype (Figure 1B,F), which could be used to study the photosynthesis of leaves and siliques. In this study, the candidate gene-encoded ChlH was obtained by map-based cloning, and a sequence analysis revealed a base substitution (G to A), causing an amino-acid substitution from glutamic acid to lysine in domain VI of ChlH at position 1349 (Supplementary Figure S1).

MgCH, a key enzyme in chlorophyll synthesis, catalyzes the conversion of proto IX to Mg-proto IX. MgCH contains three subunits, ChlI, ChlD and ChlH, of which ChlH contains the insertion site of the magnesium ion for the catalytic reaction. The structure and function of ChlH have been studied extensively [18,20,21,60]. However, there are few studies on ChlH in *Brassica*. We identified the ortholog of the gene in *Arabidopsis* that encodes the H subunit of MgCH as being the candidate gene in *B napus* mutant *yl1*. A single base substitution, deletion, addition, or structural variation in a gene may cause a gene mutation. Zhao et al. also found the mutation *yvl*, resulting in a chlorophyll-deficient phenotype, in the *BnaA03g04440D* gene. The gene is the same as *BnaA03G0054400ZS*. A single nucleotide substitution in *BnaA03g04440D* led to the premature termination of the expression of the gene. All amino acids after 1371 (Ser) in *yvl* were deleted, and the last α-helix structure at the C-terminus was destroyed [16]. Zhao et al. concluded that the impairment of PEP activity may lead to the impairment of chloroplast development and chlorophyll degradation in *yvl* [16]. However, we think the genes they studied were actually nuclear genes rather than plastid genes. In mutant *yl1*, the 1349th amino acid in ChlH changed from glutamic acid to lysine (E to K), but did not terminate expression prematurely (Supplementary Figure S1). The expression level of the candidate gene in *yvl* was significantly lower than that in the wild-type, but the expression level in *yl1* was not significantly different to NY18 (Figure 6). At maturity, compared with the wild-type plants, the agronomic traits of *yvl* had no significant difference in the plant height, 1000-seed weight, and other traits. However, the main agronomic traits of *yl1* were greatly affected [32]. The two mutants exhibited mutations in the same gene, but the mutation site and the expression patterns of the two mutants were quite different. There were fewer DEGs in mutant *yl1*, but it had a greater impact on agronomic traits. This indicated that the amino acid (Glu1349) located in domain VI of ChlH may be a conserved amino acid that plays an important role in the ChlH function (Supplementary Figure S1).

The ChlH, where the insertion of Mg^2+^ takes place, is the catalytic subunit of MgCH [17]; therefore, the mutation of ChlH may affect the activity of MgCH, and the synthesis of chlorophyll was blocked in *yl1*. There is much evidence suggesting that chlorophyll is needed for the stabilization of LHC [61,62,63]. In the research of the *chlorina f2* mutant lacking chlorophyll *b* of barley, the mutant contained mRNA for membrane polypeptides present in the mutant at levels comparable to those of wild-type as determined by the uptake assay; the absence of chlorophyll *b* caused these polypeptides to turn over rapidly [63]. In *yl1*, the results of RNA-seq showed there were no significant changes in the gene expression levels of most LHC subunits. However, *Lhcb2.4* was significantly downregulated, and Zhao et al. also found that *Lhcb2.4* reduced in *yvl* (Supplementary Table S7) [16]. There could be some mechanism that specifically downregulates *Lhcb2.4*. ChlH is not only a key gene in the chlorophyll synthesis pathway, but is also involved in the transmission of the plastid-derived signal. Under normal circumstances, the *gun* mutation causes an abnormal accumulation of *Lhcb* genes. Kindgren et al. found that the repression of *Lhcb* following a high-light treatment in *gun5* was similar to the repression observed in the wild-type [63]. Therefore, we speculated that the light conditions when sampling may have caused the abnormal decrease in the expression of *Lhcb2.4*. The results of structure prediction showed that the secondary structure of the mutant site did not change (Supplementary Figure S2B). There were no significant expression differences of ChlH. Mutations may affect the stability of ChlH. The *GUN5* gene, which encodes ChlH, was identified as a component of the plastid-to-nucleus signal transduction [21]. Previous studies have shown that *GLK* genes were responsive to plastid retrograde signals and helped to coregulate and synchronize the expression of a suite of nuclear photosynthetic genes [64]. *Golden 2-like* regulates chloroplast development in *Arabidopsis*, and *GLK* genes strongly regulate ChlH, the *LHC* super-gene family, and other genes involved in chlorophyll biosynthesis [64]. The results of RNA-seq showed that the transcript level of *GLK1* significantly decreased, and *GLK2* decreased slightly (Supplementary Table S7). We speculated that the instability of LHC complexes without chlorophylls would be the primary cause of the impaired chloroplast development in this mutant, and the regulation of plastid signaling may have an additional effect. In *yl1*, the expression of *CLH2* involved in chlorophyll decomposition was significantly increased, which aggravated the chlorophyll deficiency phenotype.

A large number of genes have been mapped using simple sequence repeats [57], in/Dels [55,57], amplified fragment length polymorphisms [65], and other marker systems. The analysis by DNA electrophoresis, a process which is very time-consuming, is needed when ordinary molecular markers are applied to plant breeding and gene mapping. Moreover, compared to SNP markers, traditional molecular markers have the disadvantages of fewer markers and an uneven distribution. Based on SNPs, researchers have developed competitive allele-specific PCR [66], PARMS [39], and other genotyping techniques. High-throughput genotyping can be carried out by scanning the fluorescence type, eliminating the need for DNA gel electrophoresis and other complex operations. At the same time, researchers also developed a method that can be genotyped by ordinary PCR in the laboratory, based on SNP loci [33]. In the present study, PARMS SNP markers were designed to fine map the candidate gene. To facilitate researchers to use this germplasm resource, a PARMS functional marker BnaYL1 for mutation sites in *yl1* was developed, and the test results showed that BnaYL1 was completely cosegregated with the chlorophyll-deficient phenotype in different populations.

## 5. Conclusions

In our previous study, a chlorophyll-deficient mutant, *yl1*, was obtained by EMS mutagenesis, and the candidate gene was fine-mapped to an interval of 304.7 kb on chromosome A03 by QTL-Seq and map-based cloning strategies. Within this interval, 58 annotated or predicted genes were identified in “ZS11” reference genomes. Combining the results of the functional annotation analysis, transcriptome analyses, expression analysis, and sequence variation analysis, *BnaA03G0054400ZS*, which encodes the H subunit of MgCH, was considered the most likely candidate gene. An SNP in the fifth exon of *BnaA03G0054400ZS* caused an amino-acid substitution (Glu1349Lys). This mutation may affect the stability of CHLH, thereby affecting the expression of genes involved in the regulation of chloroplast development and chlorophyll degradation. Based on the SNP, a function molecular marker BnaYL1 was developed, which was completely cosegregated with the chlorophyll deficiency phenotype in two different F_2_ populations. These results enhanced our understanding of chlorophyll synthesis in *B. napus*.

## Figures and Tables

**Table 1:** The primer sequences used in this study. During the qRT-PCR experiment, primer 1 was used to amplify the target gene, and primer 2 was the primer of the reference gene. Primer 3 was used to clone the cDNA of NY18 and *yl1*. Primer 4 was the primer sequence of functional marker BnaYL1.

No.	Forward Primer Sequence (5′–3′)	Reverse Primer Sequence (5′–3′)	Purpose
1	GATCGCGCTTGTGCTTTGGG	TCGGCCTTCCAAGCTCCTCT	qRT-PCR
2	CAGGAATCGCTGACCGTAT	TCTCCCTTTGAAATCCACAT	
3	ATGGCTTCACTTATGTATTCACC	CTATCGATCGATCCCTTCAA	Cloning cDNA
4	GAAGGTGACCAAGTTCATGCTGGAAGATGATCCAGACTTTCTTGG	CCTTCAATCTTGTCTTCAACCTGG	Molecular marker
	GAAGGTCGGAGTCAACGGATTAGGAAGATGATCCAGACTTTCTTGA		

## Data Availability

The raw sequence data were deposited in the NCBI (https://www.ncbi.nlm.nih.gov/sra/ (accessed on 18 January 2022)) Sequence Read Archive (SRA) under BioProject PRJNA804373, with accession numbers SRR17931830, SRR17931831, SRR17931832, SRR17931833, SRR17931834, and SRR17931835. All other data analyzed during this study are included in this published article and its Supplementary Information Files.

## Supplementary Materials

The following are available online at https://www.mdpi.com/article/10.3390/biom12030402/s1, Figure S1: the amino-acid sequence difference of protein encoded by *BnaA03G0054400ZS* gene between NY18 and *yl1*, Figure S2: the prediction of protein spatial structure of the mutation sites, Table S1: primer sequences of PARMS markers used for fine mapping the *BnA03.Chd* locus, Table S2: the PCR reaction system of candidate gene cloning, Table S3: summary of resequenced data of the two parents (NY18 and *yl1*) and the two F_2_ bulks (G- and Y-pool), Table S4: seventy-six recombinants and their genotypes detected by marker M1 and M9 in 1524 chlorophyll-deficient individuals of (Holly × *yl1*) F_2_ population, Table S5: summary of transcriptome data of leaves from wild-type NY18 and mutant *yl1*, Table S6: the expression level of all genes in the chlorophyll synthesis pathway, Table S7: transcriptional changes of differential expression genes between NY18 and *yl1* based on RNA-seq data, Table S8: differentially expressed genes in different enriched KEGG pathways, Table S9: the annotated genes in *BnA03.Chd* interval of ZS11 genome, Table S10: the specific data and statistical analysis results of qRT-PCR of NY18 and *yl1*.

## Abbreviations

ABA, abscisic acid; ALA, 5-aminolevulinic acid; ANOVA, analysis of variance; ChlD, D subunit of MgCH; ChlH, H subunit of MgCH; ChlI, I subunit of MgCH; ChlM, magnesium protoporphyrin IX methyltransferase; DEGs, differentially expressed genes; EMS, ethyl methane sulfonate; GluTR, glutamyl-tRNA reductase; GLK, Golden 2-like; MgCH, magnesium chelatase; LSD, least significant difference; Mg-Proto IX, magnesium protoporphyrin IX; NY18, Ningyou18; PARMS, penta-primer amplification refractory mutation system; PEP, plastid-encoded RNA polymerase; Proto IX, protoporphyrin IX; QTL, quantitative trait loci sequencing; SD, standard deviation; SNPs, single nucleotide polymorphisms; ZS11, Zhongshuang11.
