# Supplementary figures and images for: Fine Mapping and Characterization of a Major Gene Responsible for Chlorophyll Biosynthesis in Brassica napus L
Source: Biomolecules. 2022 Mar 4;12(3):402. doi: 10.3390/biom12030402 (PMC8945836; doi:10.3390/biom12030402)

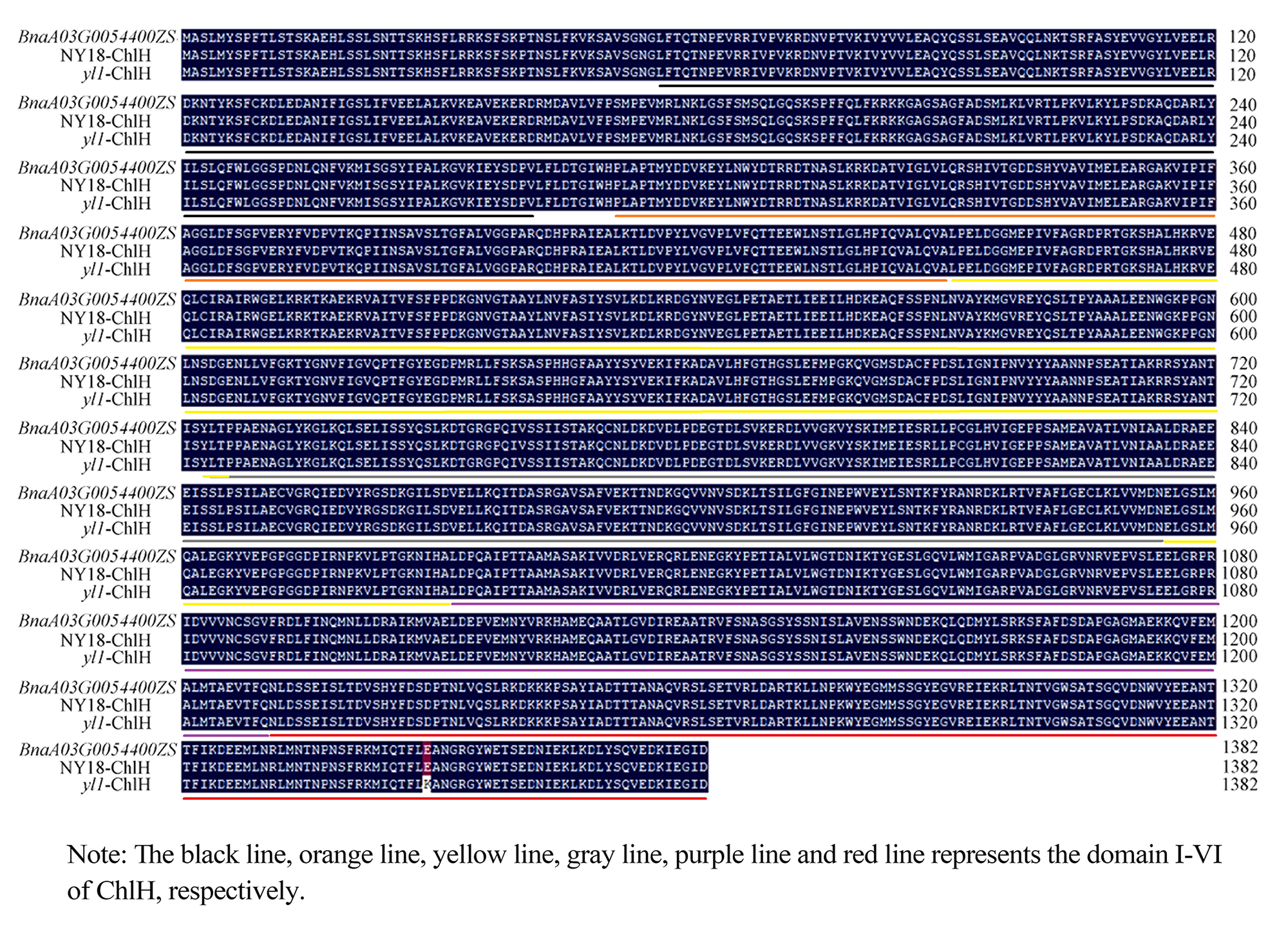

Supplement: Supplementary file 1 [file biomolecules-12-00402-s001.zip › Supplementary Figure S1.tif]

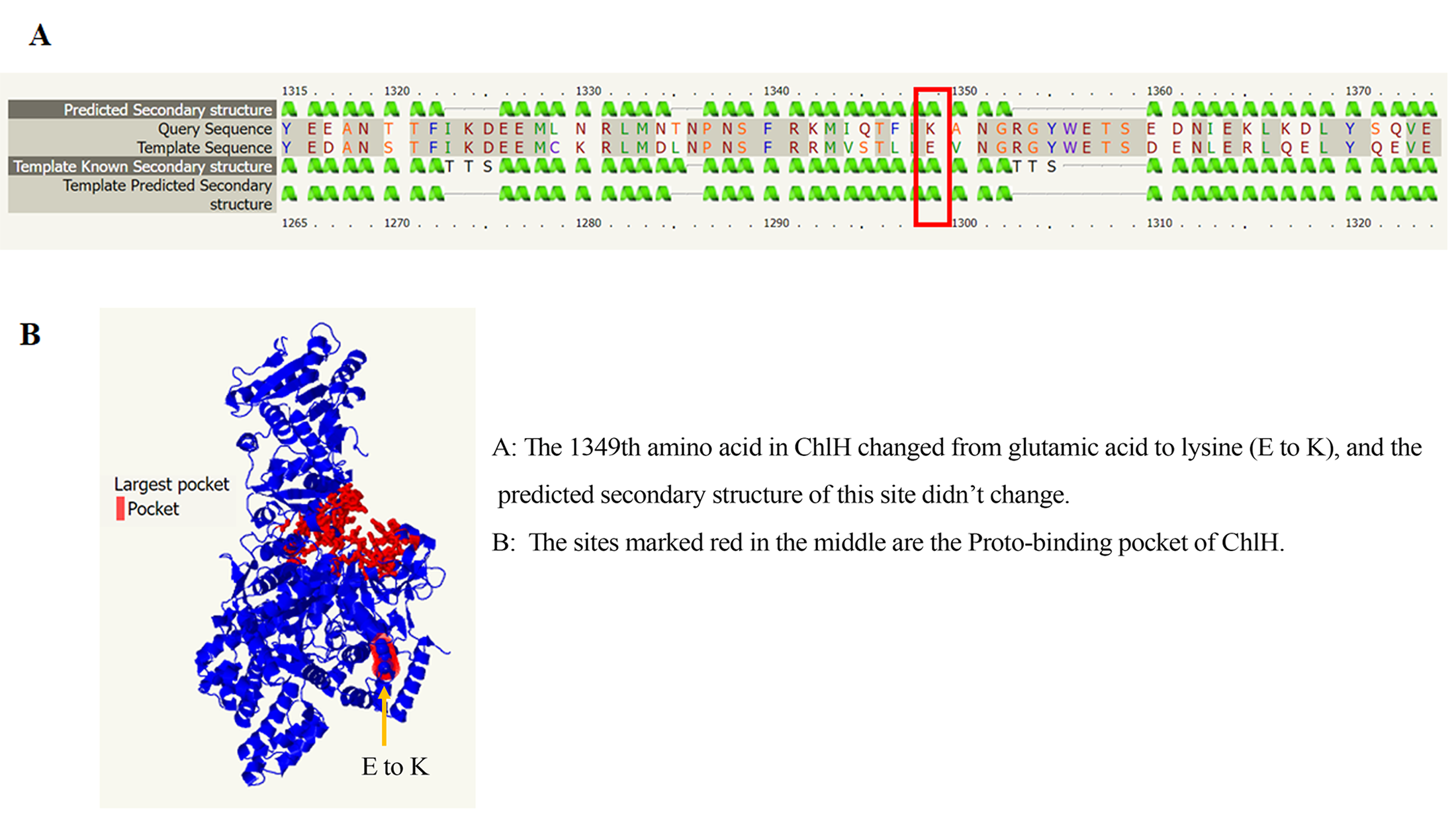

Supplement: Supplementary file 1 [file biomolecules-12-00402-s001.zip › Supplementary Figure S2.tif]
